# Guidelines for Prehospital Management of Traumatic Brain Injury 3rd Edition: Executive Summary

**DOI:** 10.1227/neu.0000000000002672

**Published:** 2023-09-26

**Authors:** Gregory W. J. Hawryluk, Al Lulla, Randy Bell, Andy Jagoda, Halinder S. Mangat, Bentley J. Bobrow, Jamshid Ghajar

**Affiliations:** * Neurological Institute, Cleveland Clinic, Akron General Hospital, Fairlawn, Ohio, USA;; ‡ Brain Trauma Foundation, Palo Alto, California, USA;; § Department of Emergency Medicine, UT Southwestern Medical Center, Dallas, Texas, USA;; ‖ Uniformed Services University of Health Sciences, Avera Brain and Spine Institute, Sioux Falls, South Dakota, USA;; ¶ Department of Emergency Medicine, Mount Sinai, New York, New York, USA;; ** Department of Neurology, University of Kansas Medical Center, Kansas City, Kansas, USA;; ‡‡ Department of Emergency Medicine, McGovern Medical School at the University of Texas Health Science Center at Houston (UT Health), Houston, Texas, USA

**Keywords:** Guidelines, Prehospital, Brain Trauma Foundation, Recommendations, Brain injury, Head injury, Emergency, Ambulance, First responder

## Abstract

Prehospital care markedly influences outcome from traumatic brain injury, yet it remains highly variable. The Brain Trauma Foundation's guidelines informing prehospital care, first published in 2002, have sought to identify and disseminate best practices. Many of its recommendations relate to the management of airway, breathing and circulation, and infrastructure for this care. Compliance with the second edition of these guidelines has been associated with significantly improved survival. A working group developed evidence-based recommendations informing assessment, treatment, and transport decision-making relevant to the prehospital care of brain injured patients. A literature search spanning May 2005 to January 2022 supplemented data contained in the 2nd edition. Identified studies were assessed for quality and used to inform evidence-based recommendations. A total of 122 published articles formed the evidentiary base for this guideline update including 5 providing Class I evidence, 35 providing Class II evidence, and 98 providing Class III evidence for the various topics. Forty evidence-based recommendations were generated, 30 of which were strong and 10 of which were weak. In many cases, new evidence allowed guidelines from the 2nd edition to be strengthened. Development of guidelines on some new topics was possible including the prehospital administration of tranexamic acid. A management algorithm is also presented. These guidelines help to identify best practices for prehospital traumatic brain injury care, and they also identify gaps in knowledge which we hope will be addressed before the next edition.

**ABBREVIATIONS:**
BTF: Brain Trauma Foundation
EMS: Emergency Medical Service
EPIC: Excellence in Prehospital Injury Care
RSI: rapid sequence intubation
SpO_2_: oxygen saturation
TBI: traumatic brain injury
TXA: tranexamic acid.

The Brain Trauma Foundation's (BTF's) Guidelines for the Management of Severe Head Injury, published in 1996,^1^ were the first clinical practice guidelines to be published by a surgical specialty. Implementation of these guidelines has been repeatedly associated with a 50% reduction in mortality^2^ as well as reduced cost of care.^3,4^ The BTF next created prehospital guidelines, reflecting the understanding that care and processes of care at the scene of injury, during transport, and early after arrival to the hospital markedly influences the ultimate outcome of brain-injured patients.^5,6^ Indeed, much of the benefit inherent to the BTF guidelines is attributed to improved resuscitation and the avoidance of secondary insults such as hypotension and hypoxia.^7,8^ The first BTF Prehospital Guidelines were published in 2002^5^ and the second edition followed in 2008.^6^

The efficacy of the BTF's Prehospital Guidelines 2nd edition was recently examined in the large prospective Excellence in Prehospital Injury Care (EPIC) study.^9,10^ This study included more than 130 emergency medical services and systems in Arizona and examined outcomes of patients with traumatic brain injury (TBI) before and after state-wide implementation of the BTF Prehospital Guidelines. It included 21 852 patients accrued over an 8-year period. This study found that implementation of the Prehospital Guidelines was associated with a 1.7x greater odds of surviving to hospital admission as well as 2x greater odds of survival until discharge.^9^ In intubated patients, prehospital guideline compliance was associated with a 3x greater chance of survival to discharge.^9^ Remarkably, however, variability in contemporary American prehospital TBI care remains problematic.^11^ Here we provide an executive summary of the new 3rd edition Prehospital Guidelines^12^ suitable for neurosurgeons in hopes it will promote dissemination of best practices and further improve the outcome of TBI victims.

## METHODS

### Expert Workgroup and Topic Refinement

Twenty-three panelists were selected and screened for conflicts of interest. Using a Population, Intervention, Comparator, Outcomes, Timing framework, the workgroup specified key clinical questions pertaining to both adult and pediatric populations for (1) Assessment, 2) Treatment, and 3) Transport and Decision-Making.

### Inclusion/Exclusion Criteria

Study inclusion criteria were as follows: human subjects, traumatic brain injury, English language, ≥25 subjects, randomized controlled trials, cohort studies, case-control studies, case series, databases, and registries. Exclusion criteria included inappropriate independent or dependent variables, statistics inappropriate to the research design, variables and/or sample size, case studies, editorials, comments, and letters.

### Literature Search Strategy and Evidence Review

The systematic review was registered (PROSPERO CRD42021269941) and conducted based on established methods and reporting standards.^13-16^ Ethics approval and patient consent were not required for this review of published studies. The literature search examined publications from May of 2005 through January 2022. Search strategies included Ovid MEDLINE and Cochrane databases for published literature and ClinicalTrials.gov for ongoing/completed trials. Electronic searches were supplemented by workgroup recommendations and cross-referencing.

### Data Synthesis, Quality Assessment, and Classification of Evidence

Predefined criteria based on those developed by the US Preventive Services Task Force, the National Health Service Centre for Reviews and Dissemination, and the Cochrane Collaboration were used to assess the quality of the included studies. The quality of evidence for each topic was assessed using the standards established by the Agency for Healthcare Research and Quality Evidence-Based Practice methods guidance, including study limitations, consistency, directness, and precision. The strength of the body evidence was classed as high, moderate, low, or insufficient.

### Recommendations

Recommendations were categorized for strength and quality of evidence. The strength of the recommendation was derived from the overall quality of the body of evidence used to assess the topic. Consistent with methods generated by the Grades of Recommendation, Assessment, Development, and Evaluation working group, recommendations were categorized as either strong or weak, reflecting the degree of confidence that the favorable effects of recommendation adherence outweigh the unfavorable effects.

## RESULTS

A total of 122 published articles formed the evidentiary base for this guideline update.^12^ For the included topics, 5 studies provided Class I evidence, 35 provided Class II evidence, and 98 provided Class III evidence. Sixteen manuscripts provided evidence for more than 1 topic. From this evidence, 40 evidence-based recommendations were generated, 30 of which were strong and 10 of which were weak. This contrasts with the second edition in which all recommendations were graded weak. Consistent with the structure of the preceding 2nd edition, recommendations were grouped into topics including *Assessment*, *Treatment*, and *Transport Decision-Making*.

**FIGURE 1.**
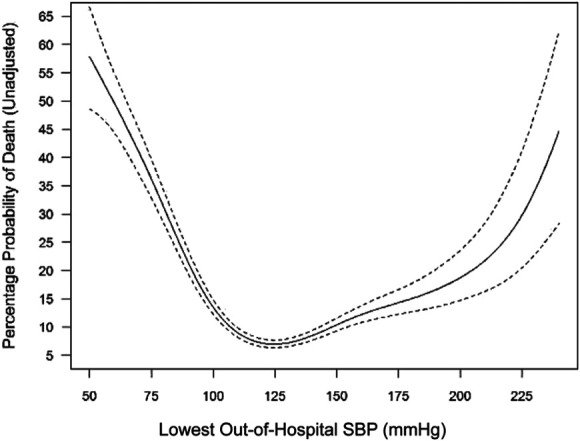
Key evidence supporting a higher SBP threshold for hypotension. Unadjusted analysis of probability of death by SBP. Unadjusted analysis of the probability of dying in the hospital plotted against lowest out-of-hospital SBP. The dotted lines represent 95% confidence bands. *Reprinted from “Annals of Emergency Medicine,” 80 (1). Spaite DW, et al. “Optimal Out-of-Hospital Blood Pressure in Major Traumatic Brain Injury: A Challenge to the Current Understanding of Hypotension,” pages 46–59, 2022, with permission from Elsevier*. SBP, systolic blood pressure.

**FIGURE 2.**
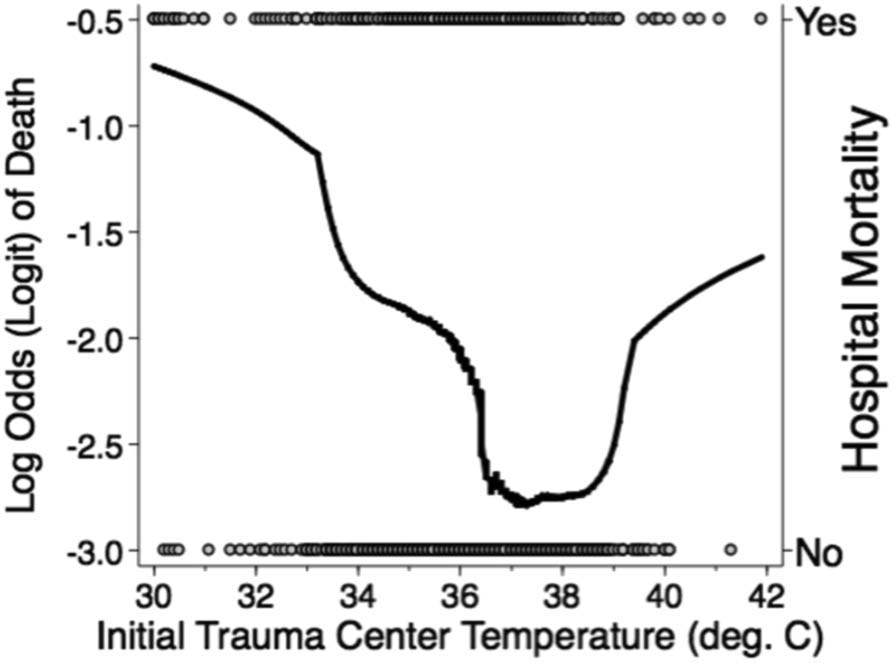
Key evidence supporting maintenance of euthermia. Lowest smoothing function for unadjusted mortality vs initial trauma center temperature. *Reprinted from “Body Temperature after EMS Transport: Association with Traumatic Brain Injury Outcomes,” Gaither JB, et al, Prehospital Emergency Care, copyright © 2017 National Association of EMS Physicians, reprinted by permission of Informa UK Limited, trading as Taylor & Francis Group*, www.tandfonline.com on behalf of 2017 National Association of EMS Physicians.

### Assessment

The Assessment section was subdivided into 3 subtopics including *Oxygenation, Blood Pressure, and Temperature*; *Glasgow Coma Scale*; and *Pupil Examination* (Table 1). The new *Oxygenation, Blood Pressure, and Temperature* section contains 13 recommendations and subrecommendations. In general, these recommendations suggest frequent or continuous monitoring of these physiologic variables. They recommend using appropriately sized equipment and target values with pediatric patients. Nine of the new recommendations are strong while 4 are weak. A new weak recommendation now suggests that temperature be measured in the prehospital environment and that euthermia be maintained (98-99°F or 36-37°C). Although the oxygen saturation threshold of 90% remains unchanged in the third edition, all adult and pediatric blood pressure targets have been increased to higher threshold values. For adults, the new recommendation is to maintain systolic blood pressure (SBP) >110 mm Hg. There are new recommendations to avoid hyperventilation by maintaining end tidal CO_2_ values between 35 and 45 mm Hg (4.6-6.0 kPa). Recommended pediatric blood pressure cuff sizes are now presented.

**TABLE 1.** Recommendations Related to the Prehospital Assessment of Brain Injured Patients

Recommendation	Strength of recommendation
Oxygenation, blood pressure, temperature	
**Patients with suspected TBI should be carefully monitored in the prehospital setting for hypoxemia (<90% arterial hemoglobin saturation), hypotension (<100 mm Hg SBP), hypertension (150 mm Hg SBP or higher), hyperventilation (end tidal CO_2_ reading between 35 to 45 mm Hg), and hypothermia or hyperthermia**	**Strong**
Optimal pediatric-specific SBPs following TBI should be targeted to the 75th and greater percentile for age 28 days and younger, >70 mm Hg 1–12 months, > 84 mm Hg 1-5 years, > 90 mm Hg 6 years and older, >100 mm Hg Adults, 110 mm Hg and above	Weak
While no specific data exist for hard cutoff values, optimal adult specific SBPs following TBI are dependent on a variety of factors and should be targeted to 110 mm Hg or greater as lower values are associated with worse outcomes. Optimal targets may be higher	Weak
**Blood oxygen saturation should be continuously measured in the prehospital setting with a pulse oximeter and supplemental oxygen administered to maintain blood oxygen saturation above 90%**	**Strong**
**Appropriately sized pediatric oximetry sensors should be used in children**	**Strong**
While no specific data exist for hard cutoff values, optimal oxygen saturation levels following TBI are dependent on a variety of factors and should be targeted to 90% or greater as lower values are associated with worse outcomes. Optimal targets may be higher	Weak
**Systolic and diastolic blood pressure should be measured in the prehospital setting using the most accurate method available and should be measured frequently (every 5-10 min) or monitored continuously if possible**	**Strong**
**Appropriately sized pediatric blood pressure cuffs should be used to measure blood pressure in children. In resource limited settings, where pediatric blood pressure cuffs are unavailable, documentation of mental status, quality of peripheral pulses, and capillary refill time should be monitored continuously as surrogate measures**	**Strong**
**Blood pressure cuffs should be matched to patients' size** **Infants—cuff size 6 × 12 cm** **Children—cuff size 9 × 18 cm** **Small adult—cuff size 12 × 22 cm** **Adult—cuff size 16 × 30 cm** **Large adult—cuff size 16 × 36 cm**	**Strong**
**Appropriately sized pediatric blood pressure cuffs should be used to measure blood pressure in children. In resource-limited settings, where pediatric blood pressure cuffs are unavailable, documentation of mental status, quality of peripheral pulses, and capillary refill time should be monitored continuously as surrogate measures**	**Strong**
**Ventilation should be assessed in the prehospital setting for all patients with an altered level of consciousness with continuous capnography to maintain end tidal CO**_**2**_ **values between 35 and 45 mm Hg**	**Strong**
Temperature should be measured in the prehospital setting and efforts should be undertaken to maintain euthermia in the patient equating to temperatures of 98–99°F/36–37°C	Weak
**In nonresource-limited settings, appropriately sized equipment to measure oxygenation, blood pressure, and temperature in children and adults should be maintained and available for routine use by trained prehospital health care professionals**	**Strong**
GCS score	
**The adult protocol for standard GCS measurement should be followed in children older than 2 years. In preverbal children, the P-GCS should be employed**	**Strong**
The GCS score should be reported every 30 minutes in the prehospital setting and whenever there is a change in mental status to identify improvement or deterioration over time. Confounders to the GCS such as seizure and postictal phase, ingestions and drug overdose, as well as medications administered in the prehospital setting that impact GCS score should be documented	Weak
**The GCS must be obtained through interaction with the patient (ie, by giving verbal directions or, for patients unable to follow commands, by applying a painful stimulus such as nail bed pressure or axillary pinch)**	**Strong**
**The GCS should be measured after airway, breathing, and circulation are assessed, after a clear airway is established, and after necessary ventilatory or circulatory resuscitation has been performed**	**Strong**
**The GCS should be measured prior to administering sedative or paralytic agents when possible and when not delaying airway stabilization or after these drugs have been metabolized as they may obscure correct scoring**	**Strong**
**The GCS should be measured by prehospital professionals who are appropriately trained in how to administer the GCS to both adults and children**	**Strong**
**The GCS of the prehospital patient, including any changes in score, should be communicated to receiving facilities during all communications and upon arrival**	**Strong**
Prehospital assessment of neurologic status using the SMS or the isolated motor component of the GCS may provide similar diagnostic and prognostic utility to the complete GCS in adults and may be used in trauma systems organized to incorporate these measures	Weak
Pupil examination	
**Pupils should be assessed in the prehospital setting after the patient has been resuscitated and stabilized, with the examination recorded and relayed to the receiving facility, for use in diagnosis, treatment, and prognosis**	**Strong**
**When assessing pupils, the following should be examined for and documented:** **Evidence of orbital and ocular trauma** **Comparison of left and right pupillary findings. Clinically significant asymmetry is defined as >1 mm difference in diameter** **Presence of unilateral or bilateral dilated pupil(s)** **Presence of fixed and dilated pupil(s). A fixed pupil is defined as <1 mm response to bright light** **Confounders to pupil examination**	**Strong**

GCS, Glasgow Coma Scale; SBP, systolic blood pressure; SMS, Simplified Motor Score; TBI, traumatic brain injury.

Strong recommendations are presented in bold. Weak recommendations are presented in unbolded font.

Reproduced from Lulla et al 2023^12^ with permission from the authors.

In this edition, 8 recommendations are provided related to application of the Glasgow Coma Scale (GCS),^17^ 6 of which were rated strong. There is a new recommendation to reassess the GCS at least every 30 minutes. Also new is the recommendation to communicate the GCS and changes in GCS during every communication with the receiving hospital's care team. A new recommendation also acknowledges the important prognostic information inherent to the motor component of the GCS which can also be assessed using the Simplified Motor Score.^18,19^

In the third edition, 2 recommendations are provided related to pupil examination, both of which were rated as strong. The new recommendations are only subtly changed, recommending communication of the pupil findings to the hospital care team as well as the identification and recording of potential confounds to the pupil examination.

### Treatment

The second edition guidelines subdivided *Treatment* into 3 parts including *Airway, Ventilation*, *and Oxygenation*; *Fluid Resuscitation*; and *Cerebral Herniation.* In the new third edition, the subtopic *Cerebral Herniation* is renamed *Hyperventilation and Hyperosmolar Therapy for Suspected Increased Intracranial Pressure* (Table 2).

**TABLE 2.** Recommendations Related to the Prehospital Treatment of Brain-Injured Patients

Recommendation	Strength of recommendation
Airway, oxygenation, and ventilation	
**All patients with suspected severe TBI should be placed on continuous oxygen supplementation via nasal cannula or face mask in the prehospital setting in order to minimize secondary insults related to hypoxia**	**Strong**
**Hypoxemia (SpO**_**2**_ **<90%) should be monitored using continuous pulse oximetry and corrected immediately upon identification by (1) ensuring appropriate airway positioning and (2) administering continuous, supplemental oxygen**	**Strong**
**If signs of hypoxia persist (central cyanosis and/or hypoxemia on pulse oximetry) despite increasing the flow and concentration of continuous supplemental oxygen, the following stepwise strategies should be undertaken with re-evaluation of oxygen saturation and respiratory effort following each strategy:** **1. airway repositioning** **2. positive pressure ventilation as with bag-valve mask ventilation in conjunction with appropriate airway adjuncts (eg, oropharyngeal airway), and/or** **3. supraglottic airway or endotracheal intubation by a trained health care professional**	**Strong**
**An airway should be established, by the most appropriate means available, in patients who have signs of severe TBI, GCS <9, or 9 and decompensating, the inability to maintain an adequate airway, or if hypoxemia is not corrected by supplemental oxygen**	**Strong**
**EMS systems implementing endotracheal intubation protocols including the use of RSI protocols should confirm endotracheal tube placement in the trachea by the presence of bilateral breath sounds on auscultation, ETCO**_**2**_ **detection, and/or capnography. Intubated patients in the prehospital setting require continuously monitored oxygenation, ETCO**_**2**_**, and frequent blood pressure monitoring**	**Strong**
**Patients requiring respiratory support with positive pressure ventilation should be maintained with normal breathing rates (approximately 10 breaths per minute with ETCO**_**2**_ **35-45 mm Hg), and hyperventilation (ETCO**_**2**_ **<35 mm Hg) should be avoided. Ventilatory adjuncts such as pressure-controlled bags, ventilation-rate timers, ETCO**_**2**_ **monitoring, and ventilators should be used to support appropriate ventilation and minimize the risk of secondary insults by avoiding hypoventilation and hyperventilation**	**Strong**
Hyperventilation and hyperosmolar therapy for suspected increased intracranial pressure	
**Hyperventilation should be avoided in the prehospital care of children and adults with TBI in the absence of signs of active cerebral herniation. Signs of active cerebral herniation include Cushing triad (hypertension, bradycardia, irregular breathing pattern), GCS < 9, posturing or lateralizing findings, progressive neurologic deterioration, and unilateral or bilateral fixed, dilated pupil(s)**	**Strong**
**Ventilation strategies should target eucapnia and avoid hypocapnia (ie, ETCO**_**2**_ **of 35-40) and be monitored using capnography**	**Strong**
**When used to address signs of active and imminent herniation, hyperventilation should target an ETCO**_**2**_ **of 30–35 mm Hg using capnography**	**Strong**
Hyperosmolar therapy (mannitol and hypertonic saline) should not be administered for the prophylactic treatment of suspected elevated ICP, with or without signs of cerebral herniation, in the prehospital setting at this time	Weak
**Prehospital administration of TXA therapy is not generally and widely indicated for the prophylactic treatment of suspected ICH or elevated ICP at this time. However, decisions by health care systems may vary and further evidence may support more general use**	**Strong**
Fluid resuscitation	
**Intravenous fluids should be administered in the prehospital setting to treat hypotension and/or limit hypotension to the shortest duration possible**	**Strong**
**Hypotensive patients should be treated with blood products and/or isotonic fluids in the prehospital setting**	**Strong**
Hypertonic fluid resuscitation may be administered to patients with a GCS <8 in whom increased ICP is suspected in the prehospital setting	Weak

EMS, Emergency Medical Service; GCS, Glasgow Coma Scale; ICH, intracranial hemorrhage; ICP, intracranial pressure; RSI, rapid sequence intubation; SpO_2_, oxygen saturation; TBI, traumatic brain injury; TXA, tranexamic acid.

Strong recommendations are presented in bold. Weak recommendations are presented in unbolded font.

Reproduced from Lulla et al 2023^12^ with permission from the authors.

In the *Airway*, *Ventilation*, *and Oxygenation* section, there are 6 strong recommendations. A new recommendation suggests placing all brain-injured patients on supplemental oxygen in the prehospital environment irrespective of their baseline oxygen saturation to reduce secondary insults related to hypoxia. Also new is the recommendation to ensure appropriate airway positioning as a means of correcting hypoxia. The recommendation to avoid routine use of paralytics to assist the intubation of spontaneously breathing patients which appeared in the second edition no longer appears in the 3rd edition. Different options for airway management are now explicitly articulated. Capnography is now recommended as an additional strategy to confirm successful placement of an endotracheal tube. Approximately 10 breaths per minute is now recommended to maintain a normal breathing rate, and ventilatory adjuncts such as pressure-controlled bags, ventilation rate timers, ETCO_2_ monitoring, and ventilators are now recommended to minimize hypoventilation and hyperventilation.

In the *Hyperventilation and Hyperosmolar Therapy for Suspected Increased Intracranial Pressure* subsection, there are now 4 strong recommendations and 1 weak recommendation. A 2-point GCS change is no longer recommended as a threshold for declaring neurological deterioration. Prophylactic hyperventilation remains discouraged and capnography is now recommended to help maintain eucapnia. While the previous edition recommended breathing rates to achieve hyperventilation, the third edition has removed these and instead recommends targeting capnography values. This section contains 2 new recommendations related to the prehospital administration of hyperosmolar therapy and tranexamic acid (TXA): prophylactic administration of hyperosmolar therapy in the absence of signs of herniation is discouraged as is prehospital administration of TXA for suspected intracerebral hemorrhage or intracranial pressure elevation.

In the *Fluid Resuscitation* section, 3 recommendations are largely unchanged from the previous edition. Those related to the administration of isotonic intravenous fluids have been upgraded to strong recommendations while the third recommendation permitting hypertonic resuscitation remains weak.

### Transport Decision-Making

In the section *Decision-Making Within the EMS System: Dispatch, Scene, Transportation, and Destination*, 6 strong and 1 weak recommendation are now provided (Table 3). Although the strength of these recommendations has increased, the content has only changed minimally. The new guidelines do provide new emphasis on the importance of comprehensive documentation of the time, assessment, and treatment provided by first responders.

**TABLE 3.** Recommendations Related to Transport and Decision-Making Within the EMS System for Brain-Injured Patients

Recommendation	Strength of recommendation
Trauma System Organization	
** All regions should have an organized trauma care system with comprehensive documentation of each encounter including time, assessment, and care provided**	**Strong**
** EMS should establish specific protocols directing destination decisions for patients with suspected TBI**	**Strong**
Direct versus Indirect Transport to a Trauma Center	
** Patients with suspected moderate-to-severe TBI should be transported directly to a facility with immediately available CT neuroimaging capabilities, prompt neurosurgical care, and the ability to monitor intracranial pressure and treat intracranial hypertension**	**Strong**
** **While direct transport to a trauma center is preferable for most patients, in the event that this transport is not possible, stabilization at a nontrauma center with subsequent transfer within an established trauma system may occur	Weak
Pediatric care	
** Pediatric patients with suspected TBI should be treated in a pediatric trauma center or in an adult trauma center with added qualifications to treat children in preference to a Level I or II adult trauma center without added qualifications for pediatric treatment**	**Strong**
** In a metropolitan area, pediatric patients with severe TBI should be transported directly to a pediatric trauma center if available**	**Strong**
Time to treatment	
** The mode of transport should be selected to minimize the time to needed definitive interventions for the patient with TBI**	**Strong**

CT, computed tomography; EMS, Emergency Medical Service; TBI, traumatic brain injury.

Strong recommendations are presented in bold. Weak recommendations are presented in unbolded font.

Reproduced from Lulla et al 2023^12^ with permission from the authors.

## DISCUSSION

### What Is New?

The third edition of the BTF's *Guidelines for Prehospital Management of Traumatic Brain Injury*^12^ updates the recommendations provided in 2008.^6^ This edition includes additional evidence published over a subsequent 17-year period. Without question, the most important difference in this update is that 75% of the recommendations are now strong—in the second edition, all of the recommendations were graded as weak.

**FIGURE 3.**
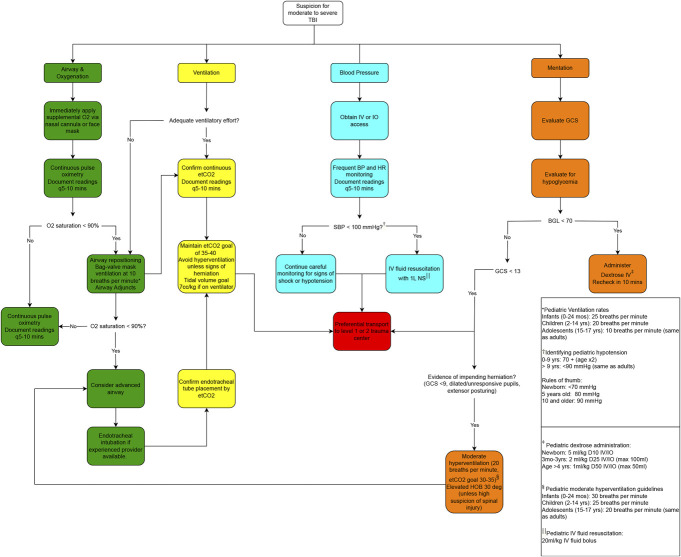
Prehospital algorithm for evaluation and management of patients with suspicion for moderate-to-severe TBI. This figure details pathways for managing the airway, breathing, and circulation in patients with suspected moderate or severe traumatic brain injury as well as a process for managing impaired level of consciousness. Key values for pediatric patients are presented. © 2023, Al Lulla. Used with permission. BGL, blood glucose; etCO2, end-tidal carbon dioxide; GCS, Glasgow Coma Scale; SBP, systolic blood pressure; TBI, traumatic brain injury.

There are additional noteworthy changes in these updated guidelines. The blood pressure targets have been increased across the age spectrum. This parallels the change in the BTF's 4th edition adult severe TBI guidelines.^20^ Immediate application of supplemental oxygen is now recommended for all patients with suspicion of moderate or severe TBI irrespective of their baseline oxygen saturation. Capnography is now more strongly and frequently recommended to guide care, especially the avoidance of hyperventilation in the absence of herniation. Recommendations related to temperature management now appear for the first time. The 3rd edition guidelines place a greater emphasis on the motor component of the GCS and no longer refer to a global 2-point change as a threshold for intervention similar to the SIBICC algorithms.^21,22^ These guidelines put greater emphasis on documentation and communication and provide more explicit, helpful recommendations related to airway management options and patient monitoring. The new edition includes a management algorithm (Figure 3); one had accompanied the first^5^ but not the second edition^6^ of these guidelines.

### Blood Pressure

Recent literature has led to important changes in our understanding of hypotension in the prehospital environment. In the past, studies investigated arbitrary cut-points leading to thresholds that were merely statistical artifacts. As well, in older literature, prehospital measures were not associated with outcome data subsequent to hospital admission. Perhaps the most important new study on this topic comes from Spaite et al^23^ who overcame these limitations in conjunction with the previously described EPIC study. In their analysis of 12 169 patients, the investigators were able to associate prehospital blood pressure measurements with outcome in a continuous fashion (Figure 1). The results strongly suggested that the traditional SBP threshold for defining hypotension—90 mm Hg—is too low. The lowest risk of mortality associated was seen when SBP was maintained above 125 mm Hg. Ultimately, the 3rd edition panelists chose to recommend maintaining SBP >110 mm Hg while acknowledging that the optimal threshold may be higher.^12^ Overall, the literature more strongly supports the avoidance of hypotension than inducing hypertension. Spaite et al^12^ and others^24,25^ have associated high blood pressures early after TBI with poor outcomes. Careful interpretation is necessary here as dropping the blood pressure in a patient with an adaptive Cushing response risks harm from cerebral hypoperfusion.

### Oxygenation

The new recommendation to provide supplemental oxygen to all patients with TBI in the prehospital environment—even those who are normoxic—aligns with growing interest in monitoring, treating, and preventing brain hypoxia after head injury. This practice was used in Arizona in conjunction with the state-wide EPIC study.^9^ It was associated with a greater likelihood of having an oxygen saturation of 100%, greater rates of reversal of hypoxemia, a lower rate of intubation and higher rate of bag valve mask-only airway management despite increased severity of both brain and overall injury in the post-intervention group. Brain hypoxia has long been understood a key secondary insult worsening outcome from TBI.^26^ It is underappreciated that brain hypoxia can induce cerebral vasodilation through autoregulatory mechanisms, contributing to intracranial hypertension.^27,28^

### Temperature

This update of the Prehospital Guidelines contains a weak recommendation to maintain euthermia—the first such recommendation in the Prehospital Guidelines. This recommendation is based on a single study published by Gaither et al^29^ (Figure 2) which was also published in conjunction with the EPIC study. Their analysis of 11 877 patients associated both low and high temperatures with worse outcome. Although this is an association that does not provide evidence of causation, it was judged sufficient to generate a recommendation. A preference for euthermia may relate to coagulopathy and predisposition to infection with hypothermia as well as increased metabolic demand and energy failure with hyperthermia.^30^

### Tranexamic Acid

TXA administration in the prehospital environment has been extensively investigated in recent years.^31,32^ Several large, high-quality studies have failed to support benefit for most brain-injured patients, leading to a strong recommendation against routine use in these guidelines. The Brain Injury: Prehospital Registry of Outcome, Treatments and Epidemiology of Cerebral Trauma study was a large prospective observational study of 1827 patients which reported increased mortality in patients treated with TXA (odds ratio 1.34, *P* < .001).^31^ The CRASH-3 study^33^ demonstrated beneficial effects of TXA only in mild and moderate TBI.^34^ The prehospital TXA for TBI trial was a randomized double-blind multicenter phase II trial performed in 20 trauma centers which demonstrated no statistical benefit with the administration of TXA.^35^ Given this, our panelists recommended that prehospital TXA not be routinely administered to TBI victims, although they allow for its use in special circumstances and care environments.

## CONCLUSION

It is generally believed that care provided early after TBI—largely aimed at resuscitating the patient and optimizing their vital signs—is more consequential for the ultimate outcome of a patient than care provided after admission to the hospital. The third edition of the BTF's Prehospital Guidelines for the Management of TBI^12^ aims to identify and disseminate best practices in prehospital care, updating the recommendations provided by the second edition 15 years ago. The updated recommendations guide patient assessment, treatment, and transport decision-making. It is hoped that implementation of these guidelines will further improve on the marked outcome benefits seen with the second edition.^9,10^ Moreover, we hope that these guidelines will draw attention to gaps in knowledge and inspire new research. Although many clinicians provide important contributions, neurosurgeons are the only physicians who can provide comprehensive care to TBI victims. It is thus important that neurosurgeons are familiar with recommendations for best practices across the continuum of care.

## COMMENTS

This publication stands as an indispensable update in the field of Traumatic Brain Injury (TBI) management, reflecting the substantial progress we have seen in evidence-based prehospital TBI care over the past 17 years. The most striking aspect of this third edition is the strengthened position of many of the previous recommendations, with 75% now classed as strong. This is a testament to the ongoing research and accumulation of high-quality evidence in this area. It offers a clear message to readers that the field is advancing and that adherence to these guidelines has the potential to significantly improve patient outcomes. The shift to maintaining systolic blood pressure (SBP) above 110 mm Hg, informed by recent studies associating a higher SBP threshold with lower mortality, presents a vital change in practice that demands clinicians' attention. The new stance on supplemental oxygen for all TBI patients, irrespective of their baseline oxygen saturation, marks a potentially lifesaving intervention. The paper successfully underscores the importance of managing and preventing brain hypoxia post-TBI, highlighting its influence on patient outcomes. The first-time introduction of a weak recommendation to maintain euthermia emphasizes the important role of body temperature management, providing a fresh angle to holistic TBI care. The clear stand against the routine use of tranexamic acid (TXA) in the prehospital environment is another evidence-based stance that aligns with recent high-quality studies, assisting clinicians in avoiding potentially harmful interventions. Finally, the explicit inclusion of a management algorithm serves as an excellent aid for prehospital personnel dealing with these complex scenarios, helping to streamline decision-making and potentially improving outcomes. In conclusion, this third edition of the guidelines represents a critical step forward in the prehospital management of TBI. The authors have carefully consolidated existing and new evidence into a set of practical, clear, and well-substantiated recommendations. They have successfully highlighted the vital role of early care in patient outcomes and set the stage for future research by identifying knowledge gaps. This paper is an invaluable resource for all those involved in TBI care and has the potential to shape clinical practice significantly in the years to come.

**Rohaid Ali**

*Providence, Rhode Island, USA*

In this well-written and informative executive summary, the authors provide a synopsis of the 3rd edition of the Brain Trauma Foundation (BTF) Prehospital Guidelines for the Management of Traumatic Brain Injury (TBI). Implementation of previous editions of these guidelines made a consequential impact on patient care in TBI, including improved survival at hospital admission and discharge time points. While neurosurgeons are not always directly involved in prehospital TBI patient care, those who are well-versed in best practices for preadmission oxygenation and ventilation, hemodynamic goals, and initial neurologic assessment will be better equipped to care for these patients in neurosurgical emergency. The updated recommendations most germane to neurosurgeons include advocating for more liberal use of supplemental oxygen and capnography (as it relates to regulating respiratory rate and preventing brain hypoxia), maintaining systolic blood pressure >110 mm Hg, maintaining euthermia, and discouraging routine administration of tranexamic acid. The authors provide detailed summaries of relevant literature to justify these guideline changes, along with a corresponding management algorithm. This executive summary can serve as a cornerstone for myriad avenues of future neurosurgical research, including the study of how implementing these guidelines will impact neurologic outcomes in TBI patients. Without question, the authors should be congratulated for distilling this important topic with clarity and attention to day-to-day practice.

**James Caruso**

*Dallas, Texas, USA*
